# Fentanyl and Sudden Death—A Postmortem Perspective for Diagnosing and Predicting Risk

**DOI:** 10.3390/diagnostics14171995

**Published:** 2024-09-09

**Authors:** Ines Strenja, Elizabeta Dadić-Hero, Manuela Perković, Ivan Šoša

**Affiliations:** 1 Department of Neurology, University Hospital Centre Rijeka, Faculty of Medicine, University of Rijeka, 51000 Rijeka, Croatia; medines4@yahoo.com; 2 Department of Psychiatry, University Hospital Centre Rijeka, Faculty of Medicine, University of Rijeka, 51000 Rijeka, Croatia; elizabeta.dadic.hero@gmail.com; 3 Department of Pathology and Cytology, Pula General Hospital, 52000 Pula, Croatia; manuela.perkovic@gmail.com; 4 Department of Anatomy, Faculty of Medicine, University of Rijeka, 51000 Rijeka, Croatia

**Keywords:** fentanyl, genetic counseling, hERG, harm reduction, KCNH2 gene, street supply, strip test, overdose

## Abstract

Sudden, unexpected deaths are extremely difficult for families, especially when the victim is a child. Most sudden deaths occur due to cardiovascular issues, and a smaller number (approximately one-quarter) are attributed to other causes, such as epilepsy. The medicinal and non-medicinal use of the synthetic opioid fentanyl, which can cause breathing problems, is frequently involved in these deaths. It is also being found more often in autopsies of sudden death cases, and the number of overdose deaths from illicit drugs containing fentanyl is increasing. There are cases in which it is mixed with other drugs. A gene known as the KCNH2 gene or human ether-a-go-go-related gene (hERG), involved in the heart’s electrical activity, can be related to abnormal heart rhythms. This gene, along with others, may play a role in sudden deaths related to fentanyl use. In response, we have examined the scientific literature on genetic variations in the KCNH2 gene that can cause sudden death, the impact of fentanyl on this process, and the potential benefits of genetic testing for the victims to offer genetic counseling for their family members.

## 1. KCNH2 Gene or Human Ether-a-Go-Go-Related Gene (hERG) in Sudden Death

**Figure 1:**
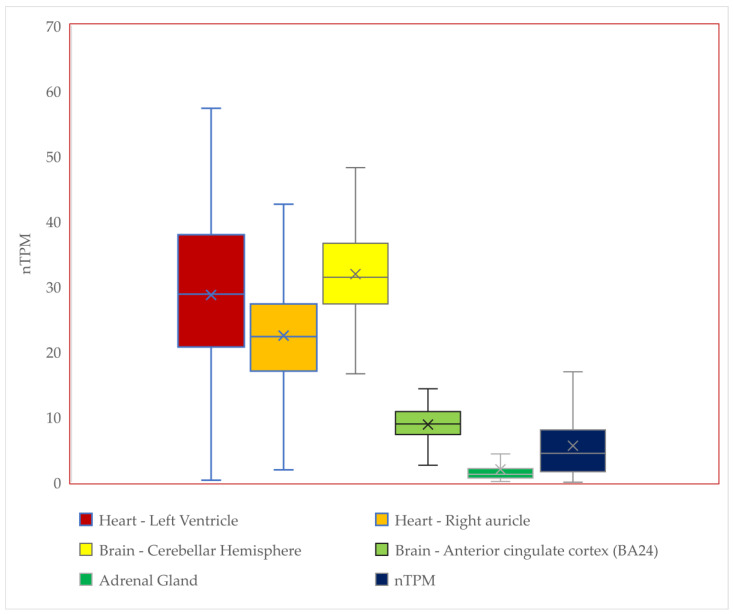
The tissue expression of KCNH2 assessed through RNA expression (normalized protein-coding transcripts per million n; TPM). Data are part of the GTEx (gene-tissue expression) RNA-seq project available at https://www.proteinatlas.org/ENSG00000055118-KCNH2/tissue/heart+muscle accessed on 17 July 2024.

Concisely, the KCNH2 gene encodes a nuclear-targeted polypeptide that mediates potassium channel gating and expression. Popularly, it is called the human ether-a-go-go-related gene (hERG). This human gene is homologous to a gene found in Drosophila melanogaster. In this species, mutations in the ether-a-go-go gene caused ether-induced leg shaking. In humans, hERG forms the major portion of one of the ion channel proteins that conduct potassium ions out of the cardiac myocytes. However, aside from that, hERG (KCNH2) is found in tissues that originate from different embryonic layers (Figure 1) [1,2].

In cardiomyocytes, neurons, or any other tissue, hERG/KCNH2 encodes an 1159-amino acid polypeptide with a characteristic K+ channel configuration. Potassium channel disorders and their discerning activation are mechanisms through which sudden death occurs [3,4]. Nevertheless, the hERG gene is responsible for the risk of drug-induced arrhythmias, which can result in sudden death (SD) [5].

The most common single-nucleotide polymorphism (SNP) to the KCNH2 gene, rs1805123 (A → C), has a minor allele frequency (MAF) of ∼24% in White Caucasian populations [6]. Another ubiquitous nonsynonymous SNP, KCNH2-K897T, appears in 30% of White Caucasians [7]. It has been identified as an intragenic modifier in some patients with long QT syndrome type 2 (LQT2); reports on its phenotypic impact and its effect on QT interval duration are discordant [8].

### Sudden Death, Sudden Cardiac Death, and Sudden Unexpected Death in Epilepsy (SUDEP)

This study defines SD as any unexpected death from natural causes. It encompasses various biological scenarios that result in rapid and unforeseen lethal outcomes. The World Health Organization (WHO) utilizes this definition, which many experts have also embraced. It refers to “death occurring within 24 h after the onset of symptoms”. It is important to note that this broad definition does not indicate a specific medical condition [9]. A comprehensive review from 2022 assigns 73% of SDs to cardiovascular causes [10]. All the other causes, including epilepsy, asthma, and intracerebral hemorrhage, account for the remaining 27%.

Sudden cardiac death (SCD) is defined as death believed to be of a cardiac cause that occurs within 1 h of the onset of cardiac symptoms or 24 h after the last witnessed distinguishable healthy report [11]. Sudden unexpected death in epilepsy (SUDEP) is a term used for the abrupt death of a patient with epilepsy when the postmortem examination fails to establish any other cause of death. Studies suggest that each year, there are about 1.16 cases of SUDEP for every 1000 people with epilepsy, although estimates vary. Cases of SUDEP are general but are not, as a rule, connected to an epileptic seizure. They occur during or immediately after it [12,13].

In the present review, we scoped the scholarly literature regarding the genetic variance of the KCNH2 gene that precipitates SD, the contribution of fentanyl to this sequence, and the feasibility and utility of providing genetic testing as a public health service.

## 2. hERG and Predicting the Risk

The human ether-a-go-go-related gene (hERG) encodes the pore-forming subunit of the gene responsible for activating potassium channels, crucial for cardiac repolarization [14,15,16]. The normal synchronous heart rhythm is controlled by a balanced flow of ions such as Na^+^, Ca^2+^, and K^+^ into and out of cardiomyocytes through ion channels. Generally, this hERG plays an essential role in relation to sudden death (SD) as potassium channels are widely distributed and critical even for setting the resting membrane potential in neurons, e.g., [17].

### 2.1. hERG in Cardiotoxicity

Safety assessment is a crucial step in the process of drug procurement. Several medications have been withdrawn from late-stage clinical trials due to their toxic effects on the heart, which are exerted through hERG-controlled potassium channels. Medications with known limitations in dose frequency for their cardiotoxicity, i.e., long QT syndrome (LQTS) are levacetalymethadol, methadone, and buprenorphine [18].

A susceptible measurement that identifies compounds exhibiting cardiotoxicity related to hERG inhibition in vivo is the hERG channel inhibition assay or hERG safety assay [19,20]. It is essential to mention that this test is an in vitro test, and not all compounds that inhibit hERG activity in vitro will proceed to cause cardiotoxicity in vivo. Take, for instance, the atypical antipsychotic medication quetiapine, which may trigger a sequence of events that turn polydrug intoxication into a fatality [21,22]. Quetiapine’s lethal effect is governed by whether some medication potentiates its inhibitory effect and, if so, to what extent [23,24].

At the same time, a novel mutation in the hERG gene has been identified and associated with azithromycin-induced acquired LQTS [14]. In the same context, the findings by Sherbini et al. connected the intake of methadone, levacetalymethadol, fentanyl, and oliceridine with the inhibition of hERG channels, prolonging the QT interval and increasing vulnerability to SCD [25].

### 2.2. Electrocardiographic Risk Predictors

Electrocardiographic predictors of sudden cardiac death were QRS ≥ 110 ms, QRST-angle > 100°, left ventricular hypertrophy (LVH), and T-wave inversions, according to the study by Terho et al. [26]. QT interval, a measure of the heart’s electrical activity, can indicate LQTS, which is associated with a high risk of SCD events [27]. Another similar condition is the rare genetic disorder called short QT syndrome (SQTS). This condition also leads to an irregular heart rhythm, shortening the time available for diastole [28,29]. It has been long demonstrated that hERG-mediated potassium channels effectively control the duration of the action potential and QT interval, which is detected in electrocardiograms [30,31]. From an electrophysiological point of view, the most relevant influence of hERG on heart rhythm is the pharmacological susceptibility of hERG to be blocked by a diversity of drugs underlying the drug-induced form of acquired LQTS with a predisposition to the Torsades de Pointes (TdP) phenomenon. Since the arrhythmogenic potential of hERG is well recognized in a drug-related context, existing preclinical guidelines require testing of all new drugs for hERG safety, typically via a hERG assay.

Conditions exist, though rare, that are characterized by a prolonged QTc interval, e.g., Romano–Ward syndrome. In some other cases, another accompanying congenital disorder, like hearing loss, is Jervell–Lange–Nielsen syndrome. Andersen syndrome is a disorder where developmental abnormalities and episodes of muscle weakness follow arrhythmia. In Timothy syndrome, lethal arrhythmias are associated with the webbing of fingers and toes, congenital heart disease, immune deficiency, intermittent hypoglycemia, cognitive abnormalities, and autism [32] (Table 1).

Integrated ECG scoring may help identify individuals requiring further measures. It can predict the risk of cardiovascular disease (CVD) in asymptomatic youth [33]. However, it is less confident in cases of subclinical CVD.

### 2.3. Harm Reduction Using Test Strips

The detection of fentanyl in drug samples, as well as the need to quickly identify toxicology-related deaths, has become pivotal in harm reduction. Relatively inexpensive immunoassays and rapid result availability make drug checking more feasible compared to lab-based methods. This may pave the way for test strips for other emerging drugs [34]. For now, it is important to understand that fentanyl test strips are designed for urine testing, primarily. Urine samples screened at autopsy may be tested using one multi-drug device and/or standalone fentanyl strip, and the strength of the color change observed correlates to the amount of drug present. Immunoassay test strips may be a helpful tool in quickly identifying toxicology-related deaths in order to triage cases and expedite turnaround times appropriately. There are commercially available over-the-counter simple urine dipstick tests [35]. Whether urine dip tests are appropriate for direct drug testing is questionable. However, non-medical fentanyl distributed on the streets is commonly mixed with xylazine, which may be unknown to the user [36]. As a result, there is a growing use of test strips specifically designed to detect xylazine along with those for detecting fentanyl [37]. However, in some cases, the effectiveness of these tests is compromised due to their known reactivity with lidocaine [38]. Testing any street-supply substance for its purity should consider that the substance is not evenly mixed with other drugs when in the form of a powder. Powders can produce the “chocolate chip cookie effect” during mixing, so it is important to test every portion of the drug [39].

The detection of the presence of illicitly manufactured fentanyl in drug samples prior to use is another form of drug testing technology. This form of fentanyl is found in the drug market in different shapes. Nasal sprays, eye drops, and drops of dried fluid on paper or a powder (sold as a powder or compressed into a pill) are all forms seen in the street supply [40]. This testing is beneficial for people who use illicit drugs, as they are at risk of fentanyl exposure.

## 3. Fentanyl and Sudden Death

**Figure 2:**
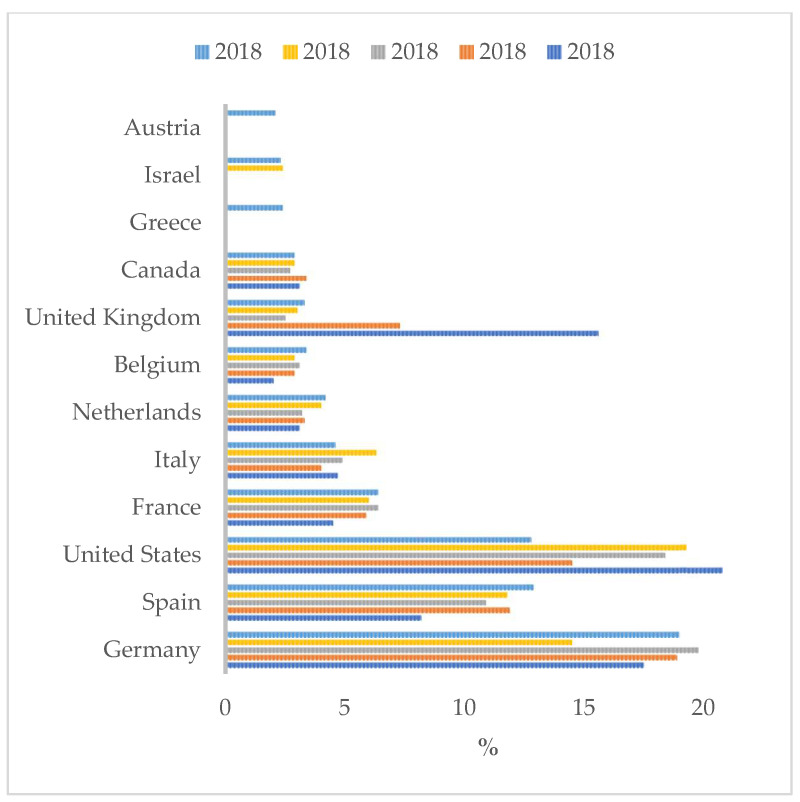
The distribution of the global fentanyl consumption (share/percentage of all non-medicinal drug use) in 2018–2022 by country (data were given for some countries by the International Narcotics Control Board (INCB); 2018–2021 values were taken from previous reports [51]).

Synthetic opioid fentanyl, which is generally used for pain management and anesthesia, is frequently abused as an illicit drug [41]. Misailidi et al. identified fentanyl’s ascension to the head of the infamous list of drug-related deaths [42,43]. Bromazolam, a benzodiazepine, and xylazine, both substances commonly mixed with fentanyl, are not affected by naloxone, a drug frequently used in fentanyl overdoses to counteract the effects of opioids like fentanyl [44,45]. While fentanyl-related deaths are a major concern in the US, with nearly 75,000 deaths in 2022, they are just beginning to increase in the European Union (EU), Figure 2 [46]. Differences in public health provisions are conjunct with patterns of use.

After non-medicinal fentanyl use began in 2012, between 100 and 150 deaths in Europe were linked to fentanyl and its derivatives in 2021 (though this number may be higher), primarily due to the diversion of fentanyl medicines rather than illicit fentanyl use [47,48]. At the same time, 70,601 people in the US died from a drug overdose that involved fentanyl [49]. Interestingly, fentanyl was reported in a toxicological analysis of a urine sample of a person who died by suicide in Croatia as early as 2007 [50].

Illicit fentanyl use poses a serious health concern, as it is associated with SD [4,52]. In addition, fentanyl significantly decreases the silencing activity of the dorsolateral pontine neurons during fentanyl-induced apnea, which underlies inadequate ventilation and respiratory depression [53,54]. Tschirhart et al. have not yet shared the information on whether cardiac electrophysiology is also disrupted and what could play a role in fentanyl-mediated SD. Currently, only a suggestion of cautious use is recommended. Then, again, fentanyl delays gastric absorption and reduces the effects of oral P2Y_12_ (chemoreceptor for adenosine diphosphate) platelet inhibitors like clopidogrel, ticagrelor, and prasugrel [55].

## 4. Improving Postmortem Healthcare

The constant and overwhelming increase in the medicinal use of prescription opioids, along with the emergence of highly potent synthetic opioids such as fentanyl, creates a risk of inducing cardiac arrhythmias and SD [56]. Thus, opioids and their combination with other drugs retain their reputation as the substances most commonly involved in drug-induced deaths. However, a share of SDs as significant as one-third to one-half involve previously healthy individuals with no morphological abnormalities [57]. The application of genetic testing as a part of postmortem procedures in managing inherited cardiomyopathies and arrhythmias is known as a “molecular autopsy”, and it involves sequencing genes implicated in cardiac channelopathies [58,59]. Moreover, depending on the literature, in 15–40% of SD cases, an external examination is not sufficient to determine a conclusive cause of death. SD with absent or minimal findings at autopsy is a red flag for genetic studies as a part of the forensic evaluation to establish the credible cause of death. This effective tool, an instrument of molecular autopsy, can be employed to uncover the cause of SD in many SD cases. In some SUDEP cases, there has been an inevitable overlap with genes involved in the pathogenic variants of cardiac-related SDs. Alternatively, for instance, there are other conditions wherein hERG mutations are conjoined with sinus bradycardia and other conditions [60,61].

Drug overdoses, as the paragons of drug-induced deaths, are highly preventable, and there is good evidence to show that interventions like avoiding mixing substances can reduce the occurrence of fatal outcomes. Unfortunately, no intervention is of solace to bereaved families, but specific autopsy findings could be red flags. In such cases, the role of genetic counseling is not substitutable. However, many forensic medicine departments lack genetic diagnostic services, so offering the complementary consultative services of a forensic medicine expert with expertise in genetics issues is wise [62,63]. Evidence of structural anomalies indicative of hypertrophic cardiomyopathy (HCM), arrhythmogenic right ventricular cardiomyopathy (ARVC), congenital anomalies of the coronary arteries, and myocarditis may be very suggestive for grieving people [64]. Such findings are intended to prompt genetic testing for identifying potential heritable causes of death, enabling the identification and evaluation of at-risk families to support grieving members.

Some estimates gauge the cause of death in about one-third of SD cases in which autopsies were otherwise inconclusive to be able to be clarified via a molecular autopsy. More recent studies suggest that the detection rate using molecular autopsy is closer to one in five [65,66]. A molecular autopsy most frequently assesses three major LQTS genes (KCNQ1, KCNH2 (simply hERG), and SCN5A) and the most common CPVT gene (RYR2) [67]. In cases of sudden deaths connected to epilepsy, variants in SCN5A, TBX18, KIF6, NEB, GUF1, TLR4, TRPM2, and PLA2G6 are all associated with SUDEP [68,69], as well as genes associated with severe epilepsies such as SCN1A and DEPDC5 [70].

In order to improve postmortem healthcare, in all autopsy-negative SD cases, molecular autopsy and comprehensive or targeted ion channel genetic testing should be considered to identify an underlying genetic cause that allows for targeted testing [58,65].

In order to proceed with the genetic investigations, an appropriate amount of multiple specimens (blood in an EDTA tube and fresh frozen tissue) from the decedent’s body at the time of autopsy must be collected. For this reason, it is necessary to be compliant with the patient’s next of kin [71,72]. Postmortem genetic testing is most likely to be indicated at the request of the decedent’s family in cases of a child’s SD. Children are more likely than adults to die suddenly without clearly suggestive anatomic findings in autopsies [65]. Information about the results and handing them to the surviving family is undoubtedly a touch-and-go moment. In general, it is essential to explain the findings to the relatives so they can understand the scenario, identify other at-risk relatives, and provide referrals for follow-up as needed.

Traditionally, tissues obtained during the autopsy are used for histological and immunohistochemical studies. These tissue samples are therefore widely accessible for re-examination. However, the DNA derived from these tissues is generally reported to be unreliable and prone to errors due to alterations in the DNA during the fixation process in formalin (i.e., formalin-fixed and paraffin-embedded tissue, FFPE). Controversies seen in the literature in this regard are hopefully waved with advanced sequencing technologies that offer advanced DNA- and RNA-purification systems [73,74].

Classically, targeted genetic testing includes even fewer genes than in Table 2. It commonly includes three genes linked to LQTS (KCNQ1, KCNH2, and SCN5A) [65,75,76]. Supplementary to this paper is Supplementary Table S1, in which a comprehensive list of genes related to SCD can be found. This was appropriate for the early years of DNA investigation, which relied on Sanger sequencing or first-generation sequencing. Such an approach soon turned out to be ineffective, with a large number of genes associated with SD recently [77,78]. To overcome the limitations of first-generation sequencing technology, advanced sequencing technology was developed. This approach is called next-generation sequencing (NGS). It helps recognize all genetic alterations associated with SD [79,80]. Since NGS allows us to perform a broader targeted gene panel testing very fast and cost-effectively, it is logical to consider a more comprehensive genomic study. With this in mind, whole-exome sequencing (WES), resulting in the identification of a great number of variants, has been introduced and will certainly improve molecular autopsy in SD cases [80].

Current cutting-edge technology in DNA sequencing, WES, detects an enormous number of variants, so it is intriguing to assess and identify the pathological significance of each variant. A certain number of these are variants of undetermined significance (VUS). Nevertheless, this is no excuse in the case when the ever-persistent dilemma of insufficient evidence regarding their pathogenicity occurs in the molecular.

## 5. Conclusions

It is recommended that all cases of SD in young individuals should involve a molecular autopsy to search for low-frequency SNPs. Molecular autopsies should identify mutations and potentially pathogenic variants in genes associated with cardiac arrhythmias (given in Supplementary Table S1), SCD, and SUDEP. Considering the significant number of low-frequency SNPs with no specific findings at autopsy and the increasing rate of fentanyl-related deaths, comprehensive genome-wide testing is advised. If the autopsy results are inconclusive, targeted gene testing for channelopathies should be performed to establish the likely cause of death. This approach can help identify at-risk relatives, potentially increasing their odds of survival and improving their quality of life.

It is important to regularly examine the KCNH2 (hERG) gene to determine the responsible party for cases of sudden death (SD). This gene is known for causing heart problems and understanding any potential risks for the person’s relatives in the future. However, it is important to be cautious if genetic testing for KCNH2 (hERG) gene variations comes back negative in SD victims, as SD is associated with more than just this one gene.

## Figures and Tables

**Table 1:** Genetic grounds and frequencies of gene variations involved in the pathogenesis of long QT syndrome (LQTS).

Topical Gene	Gene	Associated with Romano–Ward Syndrome	Associated with Jervell–Lange–Nielsen Syndrome	Associated with Andersen (A) or Timothy (T) Syndrome	Frequency (%)
	KCNQ1 (LQT1)	*	*		40–55%
*	KCNH2 (LQT2)	*			30–45%
	SCN5A (LQT3)	*			5–10%
	ANKB (LQT4)	*			<1%
	KCNE1 (LQT5)	*	*		<1%
	KCNE2 (LQT6)	*			<1%
	KCNJ2 (LQT7)			A	<1%
	CACNA1C (LQT8)			T	<1%
	CAV3 (LQT9)	*			<1%
	SCN4B (LQT10)	*			<1%
	AKAP9 (LQT11)	*			<1%
	SNTA1 (LQT12)	*			<1%
	KCNJ5 (LQT13)	*			<1%

* denotes rhe presence of a syndrome.

**Table 2:** Genes accounting for the majority of genotype-positive cases of sudden death.

Common Name		Location	Frequency of Variations in the Population with a Predisposing Condition	Phenotype	Reference
titin	TTN	2q31.2	20%	Dilated cardiomyopathy	[81]
lamin A/C	LMNA	1q22	6–8%	Cardiomyopathy, lipodystrophy, myopathy, neuropathy, progeria, bone/skin disorders, and overlapping syndromes	[82]
β-myosin heavy chain	MYH7	14q11.2	30%	Cardiomyopathy	[83]
cardiac troponin T	TNNT2	1q32.1	50–60%	Cardiomyopathy (mild or no ventricular hypertrophy)	[84]
	KCNQ1	11p15.5-p15.4	9–17%	Romano–Ward syndrome (congenital long QT syndrome)	[85]
	SCN1A	2q24.3	>10%	From self-limited and pharmacoresponsive to developmental and epileptic encephalopathies	[86]
	DEPDC5	22q12.2-q12.3	3%	A range of epilepsy syndromes	[87]

## Supplementary Materials

The following supporting information (Supplementary Table S1) can be downloaded at: https://uniri-my.sharepoint.com/:x:/g/personal/ivan_sosa_uniri_hr/ERWNLNvebAZLleFMhvP_sA8BpuzwI8ZsqiZQrQP9aZsXTg?e=H9Qbbg, accessed on 15 July 2024.
